# THE ASSOCIATION BETWEEN ACTOR-PARTNER OPTIMISM AND COGNITIVE ABILITY AMONG OLDER COUPLES

**DOI:** 10.1093/geroni/igz038.2999

**Published:** 2019-11-08

**Authors:** William J Chopik, Jeewon Oh, Eric S Kim

**Affiliations:** 1 Michigan State University, East Lansing, Michigan, United States; 2 Harvard T. H. Chan School of Public Health, Boston, Massachusetts, United States

## Abstract

Optimism has been found to be associated with physical health and interpersonal well-being. Spouses also play an important role on people’s health especially in late life. Yet, little is known about how a spouse’s optimism might be associated with an individual’s cognitive health. This study examined how actor and partner optimism in couples are associated with cognitive ability. Results showed positive associations between actor optimism and cognitive ability (.03 ≤ rs ≤ .17), and partner optimism and cognitive ability (.03 ≤ rs ≤.04), which mostly persisted over time. Further, partner optimism moderated actor optimism. Although highly optimistic people had higher cognitive ability regardless of partner’s optimism (r = .02, p = .22), people particularly benefitted from being married to an optimist (r = .05, p < .001). These results suggest that we need to consider the context of spousal relationships when understanding optimism and cognitive health in older-adulthood.

